# Roselle (*Hibiscus sabdariffa*) stem residues as a sustainable plant-based culture medium for isolation of endophytic bacteria

**DOI:** 10.1038/s41598-026-47923-7

**Published:** 2026-05-06

**Authors:** Zohor Ahmed Ibrahim, Wagdi Saber Soliman, Osama Konsowa Ahmed, Mohammed Tawfik Abbas

**Affiliations:** 1 Department of Agricultural Microbiology, Faculty of Agriculture and Natural Resources, Aswan University, Aswan, Egypt; 2 Department of Horticulture, Faculty of Agriculture and Natural Resources, Aswan University, Aswan, Egypt; 3 Department of Biochemistry, Faculty of Agriculture, Cairo University, Giza, Egypt

**Keywords:** *Hibiscus sabdariffa*, Endophytes, Rhizobacteria, Plant-based medium, Sustainable agriculture, Biological techniques, Biotechnology, Microbiology, Plant sciences

## Abstract

Roselle (*Hibiscus sabdariffa* L.) is a medicinal plant widely known for its phytochemical richness, yet its stem residues are often discarded despite potential microbiological value. This study focused on developing and optimizing a roselle stem–based plant culture medium to enhance the recovery and cultivation of endophytic bacteria associated with medicinal plants. Dried stem powders were prepared at varying concentrations (0.2, 0.5, 1, and 2 g L^− 1^) and evaluated as a nutrient source for microbial cultivation. A strain of *Bacillus sonorensis* was used to assess optimal medium concentration via optical density measurements over 240 h. The 1 g L^− 1^ concentration consistently exhibited the highest microbial growth, indicating its suitability as an alternative medium. Nutritional analysis of roselle stems confirmed the presence of key macronutrients and micronutrients supporting microbial development. The roselle-based medium recovered 43% of total colony-forming units (CFUs) compared to standard nutrient agar media. This highlights its effectiveness in cultivating microbial communities closely associated with medicinal plants. The findings suggest that roselle stems represent a sustainable, low-cost substrate for biopreparation and microbial exploration, contributing to green biotechnology and sustainable agriculture.

## Introduction

Medicinal plants have long played a significant role in human culture and healthcare, and many modern pharmaceuticals, particularly in developing countries, originate from traditional medicinal flora^1,2^. However, studies examining the relationship between medicinal plant growth and their associated microbial communities remain relatively limited^3^. The production of bioactive secondary metabolites varies considerably among plant species and strongly influences their interactions with endophytic bacteria^4–6^. Therefore, understanding how plant-associated microbial communities respond to physicochemical changes in the rhizosphere can provide valuable insights into plant–microbe interactions and ecological dynamics^7–9^.

Unlike rhizospheric microorganisms, endophytic bacteria inhabit the internal tissues of plants, where they experience a more stable and specialized environment. These bacteria are generally classified as facultative endophytes^10^ or obligate endophytes^11^, with the latter being more difficult to cultivate due to their specific nutritional requirements. Despite this specialization, endophytes are often closely associated with their host plants and rely on plant-derived nutrients for growth. Plant tissues, juices, and extracts provide essential growth factors, including vitamins, minerals, and amino acids. Consequently, plant extracts and seed-derived substrates have been successfully used as culture media for various microbial groups, including pathogenic fungi and human-associated microorganisms^12,13^.

Cultivation of endophytic bacterial communities under laboratory conditions is essential for understanding their ecological role and biotechnological potential. In recent years, plant-derived culture media have gained increasing attention as an effective strategy for mimicking the natural nutritional environment of plant-associated microbiota. Several studies have demonstrated the success of this approach using different plant species and tissues as nutrient sources. Examples include plant-based media derived from *Trifolium alexandrinum*^14^, prickly pear and *Aloe arborescens*^15^, *Hordeum murinum*^16^, *Helianthus annuus*^17,18^, *Triticum aestivum*^19^, as well as sunflower, maize, berseem clover, faba bean^20^, and *Solanum lycopersicum*^21^. These studies have shown that plant-derived substrates can effectively support the cultivation of diverse plant-associated microbial communities by providing nutrients that resemble those available in natural plant habitats.

Roselle stems represent an abundant and underutilized agricultural by-product with a distinctive nutritional and biochemical composition. They contain structural polysaccharides such as cellulose and hemicellulose, along with lignin, forming a carbon-rich biodegradable matrix that can serve as a stable substrate for microbial growth. In addition, roselle stems contain various bioactive compounds and exhibit moderate antimicrobial properties that may influence microbial selection and community structure. Their relatively high thermal stability and ordered lignocellulosic structure (crystallinity index ≈ 61.4%) further support the chemical stability of the substrate during sterilization and microbial cultivation^22^. These characteristics suggest that roselle stems may provide a suitable and sustainable plant-based medium for the recovery and cultivation of endophytic bacteria associated with medicinal plants. However, to the best of our knowledge, roselle stem residues have not previously been evaluated as a substrate for microbial culture media. Utilizing this underutilized agricultural by-product may provide a sustainable alternative to conventional synthetic media while supporting circular bioeconomy approaches in microbial biotechnology.

Therefore, *Hibiscus sabdariffa* (roselle) stems, an underutilized medicinal residue, were explored as a plant-based culture medium for isolating endophytic bacteria with plant growth-promoting traits (PGPR). The study assesses their cultivation under Aswan’s local conditions to support a cost-effective and environmentally friendly approach to sustainable agriculture.

## Materials and methods

### Roselle (*Hibiscus sabdariffa* L.) sampling plants collection

The tested host plant was the medicinal plant, roselle (*Hibiscus sabdariffa* L.), belonging to Malvaceae family (Table 1), and it was grown in the Experimental Farm, Faculty of Agriculture and Natural Resources, Aswan University, Aswan, Egypt (23°59’52"N 32°51’35"E 170 m).

**Table 1:** Roselle plant taxonomic profile: ecology, distribution, and description.

Plant name	Scientific name	Species description	Original Habitat	Geographical Location
Roselle	*Hibiscus sabdariffa*	Annual herbaceous	Tropical Africa	Southern Egypt

Scientific Classification	World Distribution	Top Producers	Distribution in Egypt	Relative importance
Kingdom: Planta Phylum: Streptophyta Class: Equisetopsida Subclass: Magnoliidae Order: Malvales Family: Malvaceae Genus: *Hibiscus* Species: *sabdariffa* https://powo.science.kew.org	Saudi Arabia, Malaysia, Indonesia, Thailand, the Philippines, Vietnam, Sudan, Egypt, Mexico, and India are home to the roselle plant^22^.	Egypt, Sudan, Mexico, Thailand, and China are the top producers of roselle flowers http://www.fao.org/	Sharqia, Minya, Qena, Luxor, Aswan, Matrouh, New Valley, Nubaria https://www.capmas.gov.eg/	Aswan: 53.22% Luxor: 43.7% Sharqia : 0.006% Minya: 0.02% Qena: 0.8% Matrouh: 0.25% New Valley: 0.023% Nubaria: 2% https://www.capmas.gov.eg/

The entire phyllosphere and rhizosphere system were sampled by separation of the vegetative parts and root system (intact roots with closely adhering soil), which was then carefully uprooted. All samples were placed in sterile, sealed bags and transported to the laboratory in an icebox. The samples were then subjected to further microbiological processing within 24 h.

### Culture media

#### Plant-only-based culture media

Roselle plant stems were prepared as dried plant powders for the preparation of plant-only growing media. To compare the nutritional properties of the tested dried plant powders, chemical analyses included total crude protein and minerals (macro- and micronutrients) were conducted by the Soils, Water, and Environment Research Institute (SWERI) of the Agricultural Research Center (ARC), Giza, Egypt.

#### Dehydrated plant powder teabags

Roselle plant stems, teabags, and culture medium were prepared according to the protocol described by^14^. The media were sun-dried for 2–3 days under direct sunlight at ambient temperature of approximately 30–40 °C until constant weight was achieved, and then oven-dried at 70 °C for 24–48 h to ensure complete moisture removal. The dried plant stems were mechanically ground and passed through a 2.0 mm sieve (mesh size verified using a standard laboratory sieve) to obtain a uniform fine dried powder. The dried roselle stem powder was extracted using distilled water as the solvent. The powdered material was soaked in distilled water at the specified concentrations (0.2, 0.5, 1, and 2 g L^− 1^) to prepare aqueous plant infusions prior to steam sterilization.

The plant-dried powders described above were used to prepare the tested culture media in their liquid form. Agar-agar (2% w/v) was added to the solid culture media. The natural pH of the roselle stem-based culture medium was measured and recorded as 6.30 ± 0.2 before sterilization at 121 °C for 20 min.

#### Growth of rhizobacteria isolates on agar plates

Pure isolates of *Bacillus sonorensis* K95 (pp. 101832), a rhizobacterium, were provided from Microbiology Department, Faculty of Agriculture, Cairo University, Giza, Egypt. The bacterial batch culture was first injected into NA (Nutrient Agar) media, and the growth and purity were assessed under a microscope. On the surfaces of agar plates that represented plant-based agar plates (HA) at different concentrations (0.2, 0.5, 1, and 2 g L^− 1^ respectively), which were made from sequential dilutions of different plant materials as well as the standard nutritional agar, aliquots of 100 µL were carefully dispersed. Scant (discontinued bacterial lawn, with dispersed colonies), good (continued bacterial lawn), and very good (continued and denser bacterial lawn) were the growth indices that were recorded after seven days of incubation at 30 °C.

#### Optimal plant medium concentration

*Bacillus sonorensis* initially inoculated in a liquid roselle medium at 30 °C for 1 week. Optical density for culture media was determined at 600 nm (OD_600_) using a spectrophotometer (SPECTROstar Nano, BMG LAbTECH GmbH, Germany) every 48 h for 7 days. All optical density measurements were performed in triplicate, and results were expressed as mean values ± standard deviation.

#### Standard chemically culture media

The standard culture media of nutrient agar^23^ were used throughout this study contained the following (g L^–1^): Beef extract, 3.0; peptone, 5.0; glucose, 1.0; yeast extract, 0.5; agar, 15; pH, 7.2.

#### Recovery of endophytic bacteria associated with roselle roots

The roots sample’s surface was decontaminated using the technique outlined by^24,25^ with some modifications for roselle plants.

Roots samples (13.05 g) were washed with running tap water to remove soil and dust and dried with filter papers. Before being chopped into tiny slices or pieces for surface sterilization, the root segments were taken to a laminar air flow hood, immersed in 75% (v/v) ethanol for two minutes, followed by two washes in sterile distilled water, a 10-minute addition of 5% (v/v) sodium hypochlorite (NaClO), and five washes in sterile distilled water. The effectiveness of surface sterilization was verified by spotting 200 µL aliquots from the final rinse water, spreading them on nutrient agar (NA) medium (HiMedia, Mumbai, India), and incubating for 24 h at 30 °C. No microbial growth was observed, confirming successful surface sterilization. Roots that had been surface sterilized were cut into 3–5 cm pieces, which were then crushed, and approximately 10 g of the sample were quantitatively added to 90 mL of a potassium chloride (0.85%) saline solution. Serial dilution was performed up to 10^− 7^ using sterile saline.

Following that, they were cultivated on 10 nutrient plates, each of which had two varieties of nutrient agar (NA) and roselle agar (HA). To count the number of bacteria in the inner coating, these plates were incubated for 24 to 72 h at 30 °C. After different bacterial colonies were chosen from the third (10^− 3^) dilution plates, the colonies were streaked onto plates, purified in fresh NA agar at least three times, and then transferred to slant agar. The purified strains were stored at -20 °C in 70% glycerol.

#### Morphophysiological identification of endophytic rhizobacteria developed on agar plates

All colony-forming units (CFU) counts were performed in triplicate plates. Representative agar plates containing 30–70 CFU per plate were selected to represent various culture media: standard nutrient agar and plant-based culture media. From these plates, pure isolates were obtained by single colony isolation. All developed colonies were examined for growth, colony and cell morphology, Gram staining, and cultural characteristics. CFU counts were expressed as log CFU g^− 1^ fresh weight.

### Screening of PGPR traits

#### Indole-related compounds production

The production test for indole-related compounds was conducted using Glickmann and Dessaux’s^26^ methods.

#### Phosphate solubilization test

Pikovskaya’s modified agar plates were used to test the phosphate-dissolving capacity^27^.

#### Potassium solubilization test

The investigated isolates were assessed using modified Aleksandrov medium in accordance with Hu et al.^28^ and Parmar and Sindhu^29^ for potassium solubilization screening.

#### Nitrogen fixing test

The micro-Kjeldahl method, as outlined by Jackson^30^, was used to determine the quantity of fixed nitrogen in the tested isolates using a modified carbon composite medium free of nitrogen (CCM) of Hegazi et al.^31^.

#### Thermophilic test

The high-temperature tolerance approach was used to conduct thermophilic testing as outlined by^32,33^.

### Molecular identification and phylogenetic analysis

The bacterial isolates were molecularly identified by partial sequencing of the 16 S rRNA gene.

Genomic DNA was extracted using a commercial DNA extraction kit according to the manufacturer’s instructions. PCR amplification of the 16 S rRNA gene (~ 1500 bp) was performed using the universal primers 27 F and 1492R. Each 25 µL PCR reaction contained PCR buffer, MgCl_2_, dNTPs, primers, and Taq DNA polymerase. The PCR conditions consisted of an initial denaturation at 95 °C for 5 min, followed by 35 cycles of denaturation at 95 °C for 30 s, annealing at 50 °C for 30 s, and extension at 72 °C for 1.5 min, with a final extension at 72 °C for 10 min.

PCR products were purified and sequenced in both forward and reverse directions using the BigDye^®^ Terminator v3.1 Cycle Sequencing Kit on an ABI PRISM 3730XL Analyzer (Macrogen Inc., Korea). The obtained 16 S rDNA sequences were analyzed using BLAST (GenBank) and EzBioCloud databases. Multiple sequence alignment was performed using SnapGene, and phylogenetic analysis was conducted using the neighbor-joining method with MEGA version 11 software.

### Data analysis

Statistical differences among treatments were evaluated using analysis of variance (ANOVA)^24^, followed by the Least Significant Difference (LSD) test at *P* ≤ 0.05^35^. Statistical analysis was performed using the R software (version 2023, R Foundation for Statistical Computing, Vienna, Austria).

## Results

### Nutritional profiles of roselle stems plant-based culture media

The nutritional composition of the examined roselle plant-only culture media was as presented in (Table 2). Stems were identified to possess numerous essential elements for the development of the examined endophyte population, including proteins, macronutrients, and micronutrients.

**Table 2:** Chemical analyses of the dehydrated powder of stems of the tested. roselle plants (*Hibiscus sabdariffa* L.).

Category	Parameter	Value
General composition (%)	Proteins	7.00
Macro-nutrients (%)	N	1.12
	P	0.07
	K	0.81
	Na	0.07
	Mg	0.16
	Ca	0.42
Micro-nutrients (ppm)	Cu	21.00
	B	9.90
	Fe	308.90
	Mn	15.10
	Se	5.40
	Pb	1.40

**Table 3:** morphological and biochemical traits of 20 endophytic bacterial isolates from roselle roots, grown on roselle stem-based medium.

S.No	Isolate code	Colony morphology						Microscopic Tests			
		Size	Color	Margins	Shape in plate	Elevation	opacity	Shape	Motility	Gram stain test	KOH test
1	HER35Z	Pinpoint	White	entire	Circular	Flat	Opaque	Cocci	+	+	-
2	HER33Z	small	White	entire	Circular	Flat	Opaque	Short rod	+	+	-
3	HER32Z	Moderate	Creamy White	entire	Circular	Flat	Opaque	Short rod	+	+	-
4	HER18Z	Moderate	White	entire	Circular	Flat	Opaque	Short rod	+	+	-
5	HER17Z	Moderate	Creamy White	entire	Circular	Flat	Opaque	Cocci	-	+	-
6	HER25Z	small	White	entire	Circular	Flat	Opaque	Cocci	+	+	-
7	HER23Z	Pinpoint	White	entire	Circular	Flat	Opaque	Short rod	+	+	-
8	HER12Z	Pinpoint	White	entire	Circular	Flat	Opaque	Short rod	+	+	-
9	HER15Z	Moderate	Yellow	entire	Circular	Flat	Opaque	Short rod	+	+	-
10	HER21Z	Moderate	White	entire	Circular	Flat	Opaque	Short rod	+	+	-
11	HER28Z	Pinpoint	White	entire	Circular	Flat	Opaque	Short rod	+	+	-
12	HER2_Y_Z	small	Yellow	entire	Circular	low convex	Opaque	Short rod	+	+	-
13	HER21_D_Z	small	White	entire	Circular	Flat	Opaque	Short rod	+	+	-
14	HER36Z	Moderate	White	entire	Circular	low convex	Opaque	Cocci	+	+	-
15	HER38Z	Moderate	White	entire	Circular	low convex	Opaque	Short rod	+	+	-
16	HER15_W_Z	Moderate	White	entire	Circular	low convex	Opaque	Cocci	-	+	-
17	HER15Z	Small	Creamy white	entire	Circular	Flat	Opaque	Short rod	+	+	-
18	HER6Z	Pinpoint	Creamy white	entire	Circular	low convex	Opaque	Cocci	+	+	-
19	HER43Z	Pinpoint	White	entire	Circular	low convex	Semitransparent	Cocci	+	+	-
20	HER46Z	Small	White	entire	Circular	low convex	Semitransparent	short rod	-	+	-

The tested Roselle stems are reported to contain 7% protein as active constituents and macro-nutrients ranged between (0.07–1.12%) and micro-nutrients (9.9–308 ppm). The plant effect was demonstrated to be relatively richer in supporting the better growth of rhizobacteria.

### Growth of rhizobacteria isolates on agar plates

**Fig. 1:**
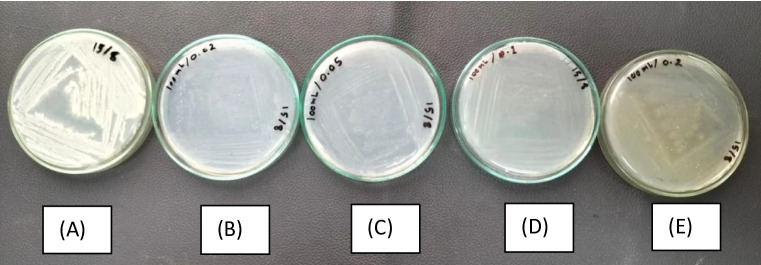
Bacterial growth on culture plant medium agar using 4 different concentrations in contrast to traditional media; (**A**) Bacterial streaked on NA agar plate, (**B**) Streaked using a plant medium with a concentration of 0.2 g L^− 1^, (**C**) Streaked on plant media using 0.5 g L^− 1^, (**D**) Streak plate on plant media using 1 g L^− 1^, and (**E**) Streaked on plant media using 2 g L^− 1^.

Agar plates made from different plant-based and synthetic standard culture media were used to examine the growth of the representative isolate, *Bacillus sonorensis* K95 (pp101832), a rhizobacterium. According to the results, the examined rhizobacteria isolate grew well on plant-based media (HA) at different concentrations (0.2, 0.5, 1, and 2 g L^-1^, respectively), especially at a concentration of 1 g L^-1^ of the plant medium (D), indicating its rich nutrient content, suitable for bacterial proliferation. In contrast, plates (B) through (E), demonstrated reduced bacterial growth (Fig. 1). A clear trend of decreasing growth with lower nutrient concentrations can be observed, with minimal to no visible growth on plates (B) and (C). These findings suggest that while stem plant- based media can support bacterial growth, optimization of concentration is essential to enhance bacterial recovery.

### Growth of rhizobacteria isolate in liquid batch cultures

**Fig. 2:**
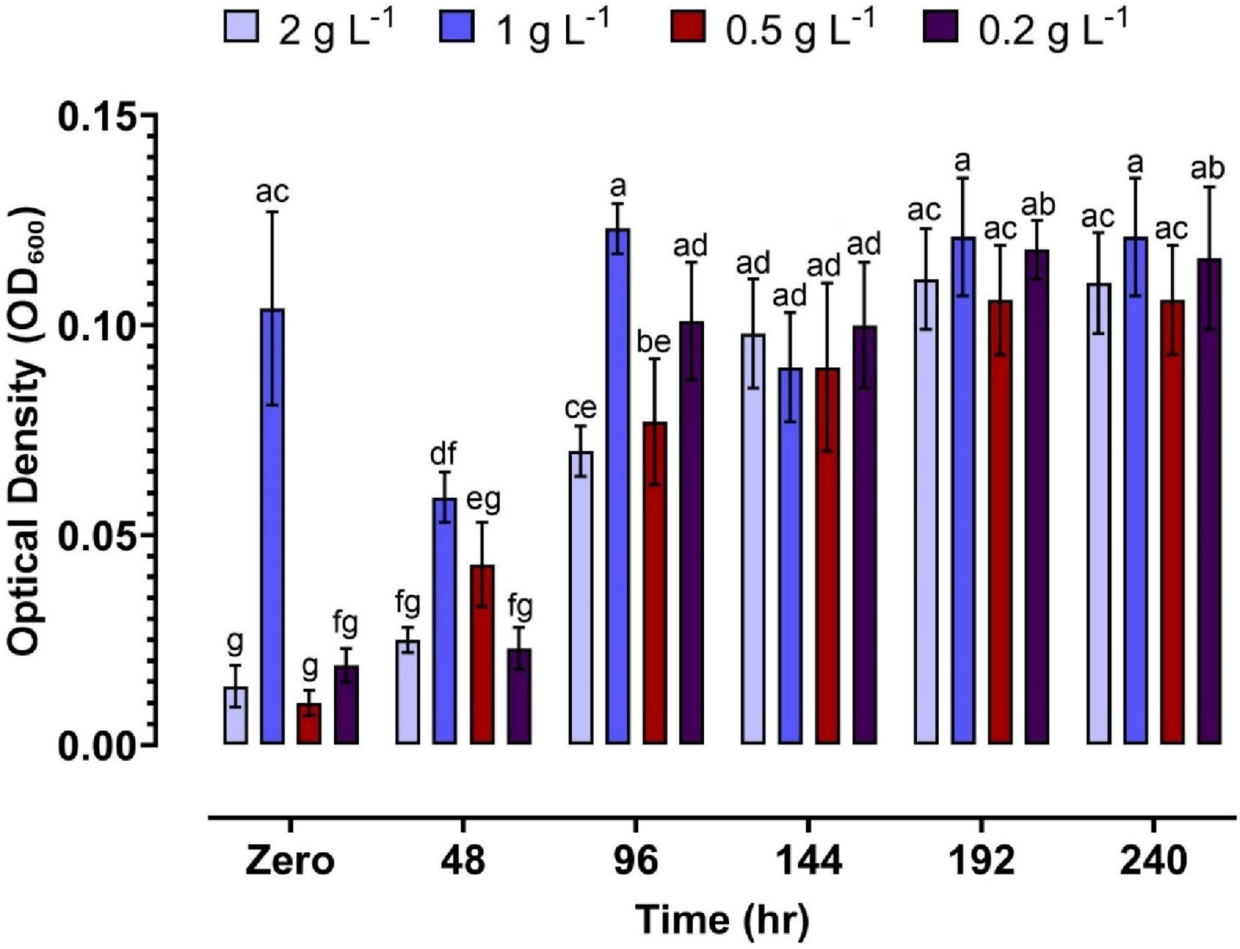
Effect of different concentrations (0.2, 0.5, 1, and 2 g L^− 1^) on optical density (OD_600_) measurements over time. Each value represents the mean of three independent replicates ± standard deviation. Different letter represent significant difference among concentration and during incubation period.

The graph illustrates (Fig. 2) the microbial growth of *Bacillus sonorensis* K95 (pp101832) response, measured as optical density at 600 nm (OD_600_), over 240 h of incubation at varying substrate concentrations (0.2, 0.5, 1, and 2 g L^-1^). The 1 g L^-1^ concentration consistently exhibited the highest growth, reaching a peak OD_600_ at 96 h and maintaining elevated levels thereafter. While 2 g L^-1^ showed a delayed response, it eventually approached similar OD_600_ values. Lower concentrations (0.2 and 0.5 g L^-1^) supported growth to a lesser extent, indicating that 1 g L^-1^ was the optimal concentration for promoting microbial biomass under the tested conditions.

### Recovery and cultivability of plant root-associated rhizobacteria on plant-based culture media

The cultivated rhizobacteria found in the root tissue of the roselle (*Hibiscus sabdariffa*) were satisfactorily sustained by the evaluated plant-based culture conditions. In comparison to the chemically synthetic standard culture media (nutrient agar), the nutrient store in the examined plant powder as such (Table 2) was sufficiently abundant to enable the growth of rhizobacteria.

**Fig. 3:**
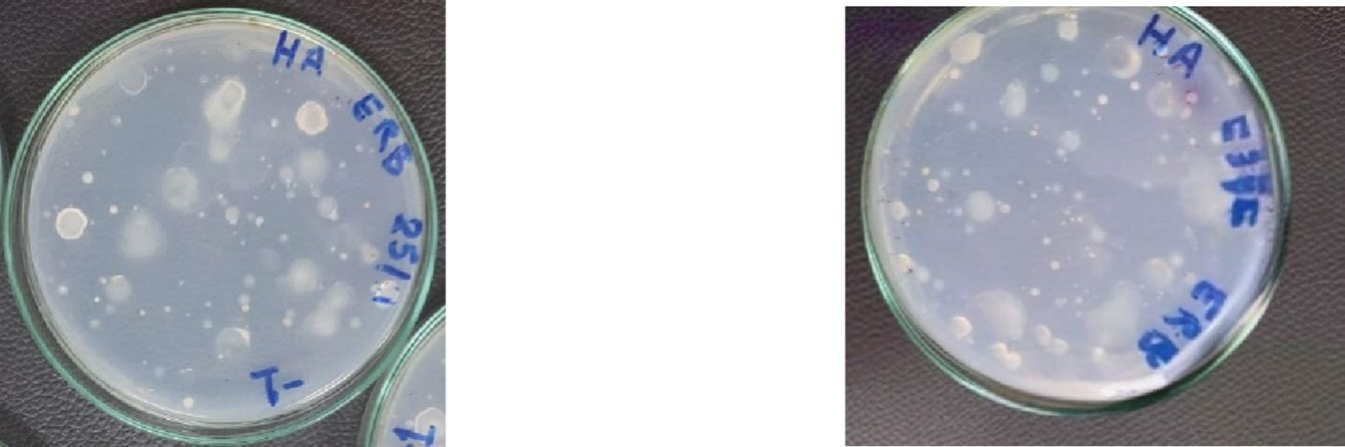
Endophytic rhizobacteria linked to plant roots are recovered on a variety of culture media. In contrast to those grown on synthetic standard culture media (nutrient agar), normally distinct colonies of rhizobacteria associated with the roots of roselle (*Hibiscus sabdariffa*) developed on agar plates of plant-based culture media prepared from 1 g of roselle stems powder.

Extending the incubation period to 7 days was found to increase the recovery of rhizobacteria, especially for the plant-based culture media, as the differences between the tested culture media decreased. Colonies that had developed were unique, easily identified, and contained rather than expanding (Fig. 3).

**Fig. 4:**
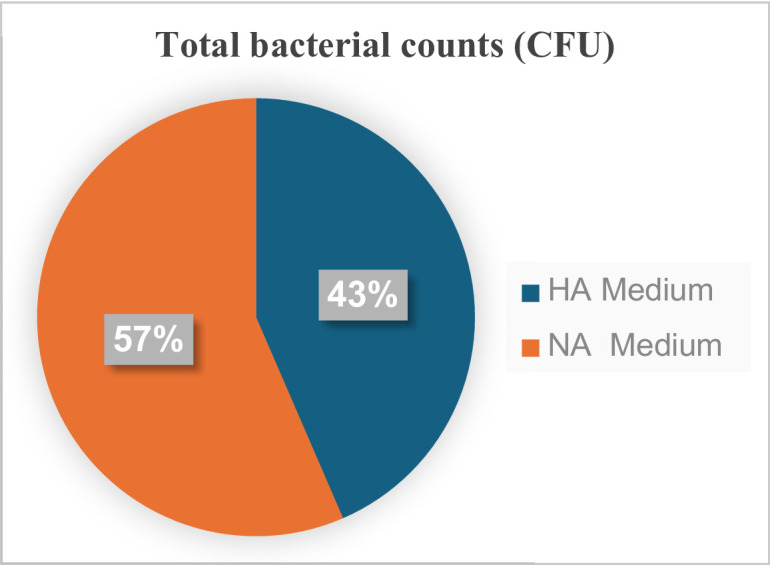
This graph shows the percentage of growth of total endophytic bacteria isolated from roselle plant roots on two types of media: roselle agar (HA) medium compared to nutrient agar (NA) medium. The percentages represent the proportion of total cultivable endophytic bacterial counts recovered on each medium.

Total endophytic rhizobacteria counts developed on log CFU counts of endo-rhizobacteria recovered by HA media, 5.5 log CFU g^-1^, while endo-rhizobacteria were 5.8 log CFU g^-1^ in NA media. In general, counts of total endophytic rhizobacteria on HA Medium were comparable with NA medium scores of 43% for the former and 57% for the latter (Fig. 4).

### Morphological growth of bacteria on different culture media: nutrient agar and roselle-based medium

The community structure of cultivable endophytic rhizobacteria was defined by further growing and characterizing isolates of these bacteria that were linked to the roots of the investigated plants and grew on representative agar plates of different culture conditions. Cultivable rhizobacteria cultivated on plant-based culture media generally had a different composition than those cultivated on regular nutrient agar.

**Fig. 5:**
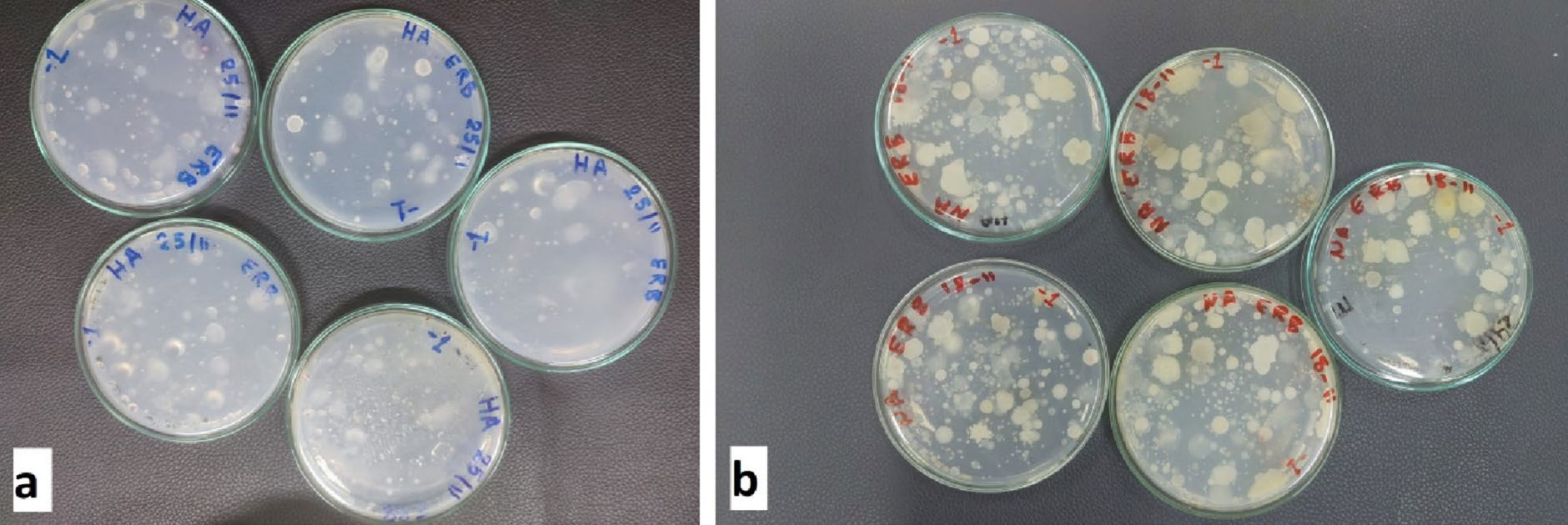
Colony morphology of bacteria isolated from endo-rhizosphere of roselle plants cultured on two types of media (**a**) roselle agar (HA) medium and (**b**) nutrient agar (NA) medium.

A total of 198 isolates were obtained as single-colonies from CFUs developed on plant-based culture (HA) and nutrient agar (NA) media, representing the total number of isolates from the endo-rhizosphere of the tested plants. Among these, 86 isolates grew on the plant-based medium, highlighting the growth on HA, while 112 isolates grew on NA. Based on the results obtained from morphological appearance, bacterial colonies grown on HA culture media were different sizes, with white transparent color and low convex, smooth texture and entire circular margin, while morphological appearance on NA culture media were different sizes and colors; some of them were flat with entire circular margin and others were low convex with irregular margins (Fig. 5).

Table 3 provides a comprehensive morphological and biochemical characterization of 20 endophytic bacterial isolates recovered from roselle *(Hibiscus sabdariffa* L.*)* roots using a roselle stem-based culture medium. Morphological traits include colony shape, elevation, margin, texture, and pigmentation features indicative of microbial diversity. Biochemical tests involve Gram staining for cell wall classification, KOH solubility for rapid Gram-type prediction, and motility assessment to detect self-propelling isolates. The observed variability highlights a rich endophytic bacterial community potentially adapted to plant-based media and may include strains with plant growth-promoting (PGPR) potential for future biotechnological applications.

Table 4 presents the 20 endophytic isolates that recorded the highest values in plant growth-promoting (PGPR) traits under in vitro conditions. In terms of IAA production, isolate HER36Z showed the highest level (37.01 mg L^-1^), followed by HER28Z (30.86 mg L^-1^) and HER23Z (30.08 mg L^-1^). For nitrogen fixation ability (N₂ assay), HER46Z recorded the highest value (35.47 mg L^-1^), followed by HER12Z (28.93 mg L^-1^) and HER43Z (30.80 mg L^-1^), indicating their effectiveness in supporting plant growth under nitrogen-deficient conditions. Regarding phosphate solubilization (PSI), isolates HER46Z and HER43Z recorded the highest values (2.61 and 2.43, respectively). For potassium solubilization (KSI), HER38Z recorded the highest value (4.03), followed by HER18Z (2.88). Most isolates exhibited growth at 45 °C, and a few showed tolerances at 50 °C. However, growth was somewhat weak or absent at 55 °C, suggesting moderate thermal tolerance. Overall, isolates HER46Z, HER36Z, HER43Z, HER28Z, and HER12Z demonstrated superior PGPR capabilities, making them promising candidates for biofertilizer development, particularly under heat stress and nutrient-poor soil conditions.

### Representative isolates and 16 S rRNA gene sequences for phylogenetic tree

The results obtained from 16 S rRNA sequencing of all 10 bacterial isolates showed similarities when compared with those of reference sequences in GenBank, and the data are presented in Table 5.

**Fig. 6:**
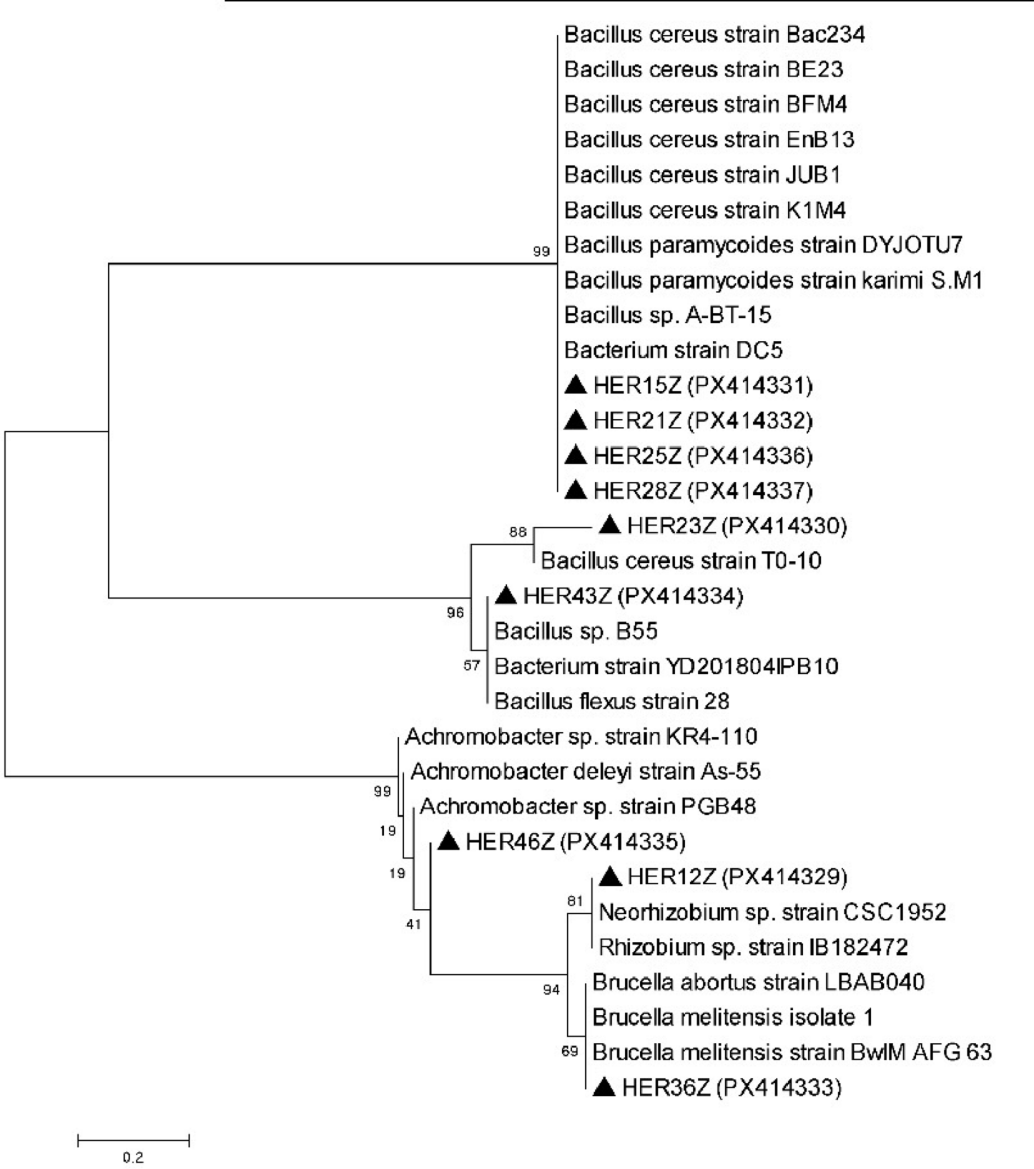
Phylogenetic tree of ten potent isolates of endo-rhizobacteria constructed based on 16 S rRNA gene sequences using Neighbor-Joining method with calculated Jukes-Cantor distances. Bootstrap values (> 50%) are shown at branch nodes. The isolates from this study are indicated in tringle bold followed by their accession numbers provided in brackets.

The 16 S rRNA gene sequences of the endophytic bacterial isolates obtained from roselle roots were analyzed, and the phylogenetic tree constructed is shown in Fig. 6. The isolated bacterial strains represented 4 different genera: *Bacillus* (60.0%), *Brucella* (10.0%), *Achromobacter* (10.0%), *Neorhizobium* (10.0%). Most of the isolated endophytic bacteria (60.0%) belonged to the taxonomic class of Bacilli. In addition, other three strains (30%) were classified as *Alphaproteobacteria* and *Betaproteobacteria*.

## Discussion

Numerous studies have demonstrated that plant-derived materials, including crude homogenates, juices, saps, and dehydrated powders, can be used directly—without supplementation—to enhance the cultivability of plant-associated microbiota^15,19–21^. In the present study, the otherwise underutilized stem residues of the roselle plant were employed as a plant-based culture medium to facilitate the recovery of previously uncultivable endophytic bacteria.

Cultivation-dependent approaches remain essential for understanding the ecology, physiology, and biotechnological potential of environmental microbiomes. However, conventional in vitro cultivation techniques typically recover less than 1% of bacteria from natural environments^20^. To address this limitation, roselle stem powder was formulated as a teabag-based medium designed to better mimic the natural habitat of the roselle microbiome, thereby enhancing microbial recovery and diversity.

An optimal stem powder concentration of 1 g L^− 1^ was identified, which yielded the highest microbial biomass. This concentration likely provides a balanced supply of carbon, nitrogen, and essential micronutrients required for bacterial growth, consistent with previous findings using clover-based plant media^14^. Despite the relatively modest nutrient content of roselle stems, a diverse community of endophytic bacteria developed without any additional supplementation. This observation supports earlier reports indicating that plant materials alone can serve as effective plant-based substrates for microbial cultivation^17^.

Endophytic bacteria isolated from roselle roots using the roselle-based medium exhibited visible growth within 72 h, comparable to growth observed on nutrient agar (NA)^16^. In contrast, isolates originating from different habitats required 7–14 days to appear, which is consistent with previous observations^15,36^. Overall, the roselle stem-based medium supported bacterial recovery and biomass production at rates comparable to those obtained with conventional synthetic media.

Bacterial counts obtained on roselle-based media represented approximately 43% of those recovered on artificial media (57%). These findings are consistent with previous reports indicating that plant-based media can yield 32–77% of total CFUs compared with 14–31% obtained using synthetic agar media^14^. The recovery rate (43%) falls within the range reported for plant-based media (32–77%), confirming the effectiveness of roselle stems as a comparable alternative substrate. Furthermore, in agreement with earlier study^37^, the inclusion of plant extracts modestly increased microbial diversity while significantly enhancing bacterial viability in both nutrient-rich and minimal media. The moderate antimicrobial properties of roselle stems may exert selective pressure, potentially inhibiting fastidious or highly specialized endophytes. This selective effect could explain differences in community composition compared to synthetic media.

Because microbial community composition is strongly influenced by host genotype and environmental conditions, the use of host-derived substrates creates a cultivation environment that favors ecologically relevant plant-associated bacteria. Such endophytes play important roles in plant growth promotion, stress tolerance, and the production of bioactive metabolites^38^. Host–microbe coevolution further shapes plant-specific microbiomes^39^, and targeting native or wild plant microbiota may offer greater functional benefits than microbial communities altered through intensive agricultural practices^40,41^.

Integrating traditional agricultural knowledge with microbiome-based strategies offers promising opportunities for improving medicinal plant productivity while maintaining biodiversity and sustainability^42^. In this context, the roselle stem-based medium proved effective in isolating endophytic bacteria exhibiting multiple plant growth-promoting traits (Table 4). Several isolates exhibited strong phosphorus solubilization (HER46Z, HER43Z), potassium mobilization (HER38Z, HER18Z), indole production (HER36Z, followed by HER28Z and HER23Z), and nitrogen fixation (HER46Z, HER12Z, HER43Z). Additionally, several isolates (HER46Z, HER36Z, HER43Z, HER28Z, and HER12Z) demonstrated tolerance to heat and other stress conditions, reflecting adaptation to the arid climate and nutrient-poor sandy soils of Aswan. These characteristics highlight their potential application in enhancing the productivity and quality of roselle and other medicinal crops^43^.

**Table 4:** The selected potent endophytes isolate recorded the highest values in PGPR traits under in vitro conditions.

S. NO	Isolates code	IAA mg L^− 1^	*N*_2_ assay mg L^− 1^	Solubilization of		Temperature tolerance ^o^C		
				*P* (PSI)*	K (KSI)**	45	50	55
**1**	HER15Z	11.13 ± 2.01 ^gh^	32.67 ± 4.28 ^ab^	2.35 ± 0.23 ^cd^	2.62 ± 0.52 ^bc^	+ve	+ve	-ve
**2**	HER21Z	16.50 ± 0.20 ^e^	-	2.61 ± 0.12 ^a^	2.67 ± 0.44 ^bc^	+ve	+ve	-ve
**3**	HER43Z	25.85 ± 2.05 ^cd^	30.80 ± 0.00 ^ab^	2.44 ± 0.11 ^abc^	2.31 ± 0.12 ^c^	+ve	+ve	-ve
**4**	HER36Z	37.01 ± 2.08 ^a^	-	2.36 ± 0.15 ^bcd^	2.57 ± 0.15 ^bc^	+ve	+ve	-ve
**5**	HER46Z	26.34 ± 2.16 ^cd^	35.47 ± 15.92 ^a^	2.61 ± 0.05 ^ab^	2.33 ± 0.14 ^c^	+ve	+ve	-ve
6	HER12Z	5.83 ± 0.31 ^ij^	28.93 ± 1.62 ^b^	2.30 ± 0.05 ^cd^	2.68 ± 0.35 ^bc^	+ve	+ve	-ve
7	HER25Z	24.46 ± 2.01 ^d^	30.80 ± 0.00 ^ab^	-	-	+ve	-ve	-ve
8	HER28Z	30.86 ± 0.20 ^b^	-	2.27 ± 0.03 ^cd^	2.25 ± 0.16 ^c^	+ve	-ve	-ve
9	HER23Z	30.08 ± 0.24 ^b^	-	-	2.39 ± 0.03 ^bc^	+ve	-ve	-ve
10	HER15WZ	3.28 ± 0.24 ^k^	-	2.45 ± 0.41 ^bc^	2.30 ± 0.18 ^c^	+ve	+ve	-ve
11	HER35Z	25.76 ± 2.01 ^cd^	-	-	2.40 ± 0.32 ^bc^	-ve	-ve	-ve
12	HER17Z	27.10 ± 0.31 ^c^	-	2.14 ± 0.05 ^de^	-	+ve	-ve	-ve
13	HER32Z	26.83 ± 2.01 ^c^	-	-	2.57 ± 0.40 ^bc^	+ve	-ve	-ve
14	HER18Z	3.61 ± 0.20 ^jk^	-	1.97 ± 0.24 ^ef^	2.88 ± 0.72 ^b^	+ve	+ve	-ve
15	HER33Z	10.55 ± 0.47 ^h^	-	2.32 ± 0.30 ^cd^	-	-ve	-ve	-ve
16	HER21DZ	1.47 ± 1.02 ^k^	-	1.88 ± 0.07 ^f^	-	+ve	+ve	+ve
17	HER2YZ	6.16 ± 2.22 ^i^	-	-	-	+ve	+ve	+ve
18	HER38Z	15.62 ± 2.01 ^ef^	-	2.22 ± 0.04 ^cd^	4.03 ± 0.25 ^a^	+ve	+ve	+ve
19	HER15DZ	14.28 ± 2.01 ^ef^	16.70 ± 0.26 ^c^	2.28 ± 0.05 ^cd^	3.70 ± 0.40 ^a^	+ve	+ve	+ve
20	HER6Z	13.39 ± 0.30 ^fg^	-	2.32 ± 0.10 ^cd^	2.21 ± 0.05 ^c^	+ve	-ve	-ve
LSD 0.05		2.26	6.21	0.25	0.50	-	-	-
Correlation coefficient (r)		-0.19	-0.51	-0.04	-0.13	-	-	-
Standard error of slope (s)		0.236	0.276	0.023	0.028	-	-	-

- ; Not detected, *PSI; Phosphate Solubilization Index, **KSI; Potassium Solubilization Index, +ve; growth, -ve; absence of growth. Different letters represent significant differences among strains.

**Table 5:** The BLAST results, including the closest species of type strain, similarity percentage, and accession number of 10 isolates obtained from roselle roots.

Isolates code	Closest relative ^a^	Acc. No	Id. (%)
HER15Z	*Bacillus sp.* A-BT-15	PX414331	99.2
HER21Z	*Bacillus cereus* strain JUB1	PX414332	100
HER43Z	*Bacillus sp.* B55	PX414334	99.64
HER36Z	*Ochrobactrum sp*. Strain QY-1	PX414333	100
HER46Z	*Achromobacter sp.* strain KR4-110	PX414335	99.28
HER12Z	*Neorhizobium sp.* CSC1952	PX414329	100
HER25Z	*Bacillus cereus* strain BE23	PX414336	99.14
HER28Z	*Bacillus paramycoides* strain karimi S.M1	PX414337	99.68
HER23Z	*Bacillus cereus* strain T0-10	PX414330	95.52
HER15WZ	-	ND	-

a: Based on the 16 S rRNA gene sequence in the database.

ND: not determined, as the sequence of this isolate was too short for database submission and getting accession number; accordingly, it is not included in the phylogenetic tree.

The proposed plant-based culture medium is safe, readily available, and cost-effective, and it supports diverse microbial communities without additional amendments. Future research should focus on the detailed chemical characterization of the medium, its influence on endophytic metabolic activity, and its scalability for bioinoculant production. Further investigation into the selective effects of roselle stem constituents on microbial community structure and function may enable targeted applications for sustainable crop improvement.

### Nutrient composition and cost comparison of nutrient agar and roselle stem–based media

A comparison of the nutrient composition of the roselle stem–based medium with conventional nutrient agar (NA) provides additional insight into its ability to support bacterial growth. Nutrient agar typically contains peptone, beef extract, and yeast extract, which supply readily available amino acids, peptides, vitamins, and minerals that promote rapid microbial proliferation. In contrast, the roselle stem–based medium is derived entirely from plant biomass and contains structural polysaccharides such as cellulose and hemicellulose, smaller quantities of soluble sugars, organic acids, and plant-derived micronutrients. Although these nutrients are generally present in more complex and less readily available forms than those in NA, they can still support microbial metabolism following enzymatic degradation by bacteria. This difference in nutrient availability may explain the slightly lower colony counts observed on the roselle-based medium compared with synthetic media, while still maintaining substantial microbial recovery and diversity. Importantly, plant-derived substrates may better mimic the natural nutritional environment encountered by plant-associated microorganisms, thereby favoring the growth of ecologically relevant endophytic bacteria that might otherwise remain uncultivable on conventional laboratory media.

To further demonstrate the economic advantage of the proposed medium, a direct cost comparison was conducted between conventional nutrient agar and the roselle (*Hibiscus sabdariffa*) stem–based medium. Conventional nutrient agar, composed of peptone, beef extract, yeast extract, and NaCl, costs approximately 13.35 EGP per liter. In contrast, the roselle stem–based medium, prepared from an agricultural by-product that is typically discarded or burned after calyx harvesting, costs only 0.14 EGP per liter, representing an approximately 98.9% reduction in cost. Beyond the substantial economic savings, this approach contributes to valorization of agricultural residues, reduces environmental pollution associated with residue burning, eliminates dependence on animal-derived components, and supports circular bioeconomy principles. Collectively, these findings demonstrate that roselle stem–based media represent a highly economical and environmentally sustainable alternative to conventional bacterial culture media.

## Conclusion

This study demonstrated the potential of roselle (*Hibiscus sabdariffa*) stems as a sustainable plant-based culture medium for the growth and recovery of endophytic bacteria. The medium prepared from stem powders not only supported the growth of a known plant growth–promoting rhizobacterium (PGPR), but also enabled the isolation of diverse endophytic bacteria from roselle roots. Among the tested concentration, 1 g L^-1^ resulted in the highest microbial biomass, indicating its efficiency as an alternative to conventional synthetic media. Owing to its favorable nutritional composition, low cost, and wide availability, roselle stem-based culture media may serve as a promising tool for microbial cultivation and biopreparation, particularly in resource-limited settings. This approach may also contribute to sustainable agricultural practices and environmentally friendly biotechnologically applications.

Nevertheless, although the roselle-based medium showed encouraging performance, further studies are needed to evaluate its suitability for cultivating fastidious or slow-growing microbial taxa and to investigate the potential influence of specific roselle-derived metabolites on microbial community composition and selection.

## Data Availability

All data generated or analyzed during this study are included in the manuscript.
